# Neuroplasticity pathways and protein-interaction networks are modulated by vortioxetine in rodents

**DOI:** 10.1186/s12868-017-0376-x

**Published:** 2017-08-04

**Authors:** Jessica A. Waller, Sara Holm Nygaard, Yan Li, Kristian Gaarn du Jardin, Joseph A. Tamm, Aicha Abdourahman, Betina Elfving, Alan L. Pehrson, Connie Sánchez, Rasmus Wernersson

**Affiliations:** 1 External Sourcing and Scientific Excellence, Lundbeck Research U.S.A., Paramus, NJ 07652 USA; 2 Intomics A/S, Diplomvej 377, 2800 Lyngby, Denmark; 3 Translational Neuropsychiatry Unit, Aarhus University, 8240 Risskov, Denmark; 4 In Vitro Biology, Lundbeck Research U.S.A., Paramus, NJ 07652 USA; 5 Center for Biological Sequence Analysis, Technical University of Denmark, 2800 Lyngby, Denmark

**Keywords:** Antidepressant, Multimodal, Synaptic plasticity, Network biology, Vortioxetine

## Abstract

**Background:**

The identification of biomarkers that predict susceptibility to major depressive disorder and treatment response to antidepressants is a major challenge. Vortioxetine is a novel multimodal antidepressant that possesses pro-cognitive properties and differentiates from other conventional antidepressants on various cognitive and plasticity measures. The aim of the present study was to identify biological systems rather than single biomarkers that may underlie vortioxetine’s treatment effects.

**Results:**

We show that the biological systems regulated by vortioxetine are overlapping between mouse and rat in response to distinct treatment regimens and in different brain regions. Furthermore, analysis of complexes of physically-interacting proteins reveal that biomarkers involved in transcriptional regulation, neurodevelopment, neuroplasticity, and endocytosis are modulated by vortioxetine. A subsequent qPCR study examining the expression of targets in the protein–protein interactome space in response to chronic vortioxetine treatment over a range of doses provides further biological validation that vortioxetine engages neuroplasticity networks. Thus, the same biology is regulated in different species and sexes, different brain regions, and in response to distinct routes of administration and regimens.

**Conclusions:**

A recurring theme, based on the present study as well as previous findings, is that networks related to synaptic plasticity, synaptic transmission, signal transduction, and neurodevelopment are modulated in response to vortioxetine treatment. Regulation of these signaling pathways by vortioxetine may underlie vortioxetine’s cognitive-enhancing properties.

**Electronic supplementary material:**

The online version of this article (doi:10.1186/s12868-017-0376-x) contains supplementary material, which is available to authorized users.

## Background

A significant challenge in major depressive disorder (MDD) is to identify biomarkers that diagnose the disease and predict treatment response. However, the heterogeneous nature of MDD makes it difficult to assign a single biomarker for these purposes. An emerging concept is that MDD may not be attributed to single genes but rather to deficits in signaling at the synapse and circuit levels, which may be reversed with antidepressant treatment. This evolving idea is the basis for the synaptogenic or excitatory synapse hypothesis of depression with a focus on specifically promoting cortical and hippocampal activation [1–4]. Several lines of evidence provide support for this idea. Postmortem studies in the prefrontal cortex of MDD patients reveal decreases in spine synapse density and reduced expression of synaptic genes, including those coding for α-amino-3-hydroxy-5-methyl-4-isoxazolepropionic acid (AMPA) subunits and neurotransmitter release machinery [5, 6]. Transcriptional repression of these genetic components is correlated with decreased dendritic complexity and depressive-like behaviors [5]. In a chronic restraint stress model of depression, glutamatergic transmission is impaired in conjunction with decreased AMPA and *N*-methyl-d-aspartate (NMDA) receptor surface expression [7]. Elevation of serotonin (5-HT) levels by antidepressant treatment can modulate glutamatergic signaling [8]. Accordingly, 5-HT signaling via 5-HT_1B_ receptors leads to potentiation of excitatory postsynaptic potentials at hippocampal synapses and increased phospho-AMPA receptor subunit levels [9].

Vortioxetine is a multimodal-acting antidepressant with a cognitive-enhancing profile. In addition to acting as a 5-HT transporter (SERT) inhibitor, it is an antagonist at 5-HT_1D_, 5-HT_3_, and 5-HT_7_ receptors, agonist at 5-HT_1A_ receptors, and partial agonist at 5-HT_1B_ receptors [10, 11]. The localization of the vortioxetine 5-HT receptor targets on glutamatergic and GABAergic neurons permits modulation of glutamatergic neurotransmission by 5-HT [8]. In support of this, vortioxetine enhances cortical pyramidal neuron activity [12] and augments theta-burst long-term potentiation (LTP), which may underlie synaptic plasticity [13]. In contrast, the selective serotonin-reuptake inhibitor (SSRI) escitalopram failed to potentiate pyramidal neuron firing [12] and LTP [13]. In addition, vortioxetine promotes dendritic branching [14], increases dendritic spine density in vivo [15], and induces spine and synapse remodeling in vitro [16], indicating a role for vortioxetine in morphological plasticity. In contrast, the SSRI fluoxetine had no effect on dendritic branching and spine density at earlier timepoints [14, 15] and failed to induce changes in spine morphology in vitro [16]. At the behavioral level, vortioxetine enhances cognitive performance in paradigms of recognition, spatial, and fear memory, and executive functioning [17–21]. In 5-HT-depleted rats, vortioxetine, but not escitalopram or duloxetine, reversed deficits in the hippocampal-dependent novel object recognition task [17] and in the Y-maze spontaneous alternation spatial memory task [21]. In middle-aged mice, a model of cognitive decline comorbid with depression, vortioxetine, but not fluoxetine, restored visuospatial impairments in the object placement test [20].

In line with these effects of vortioxetine on plasticity and cognitive performance, current studies reveal vortioxetine regulates neuroplasticity gene expression in the cortex and hippocampus, brain regions linked to cognitive dysfunction in depression [22, 23]. An acute study in adult naïve rats revealed increases in mRNA expression of genes related to serotonergic and glutamatergic signaling, protein synthesis, and dendritic spine dynamics following vortioxetine, but not fluoxetine, administration in the frontal cortex [24]. Likewise, a chronic vortioxetine study in 12-month-old mice demonstrated elevated mRNA levels of genes related to transcription, signal transduction, plasticity, dendritic spine remodeling, and neurotransmitter release in the hippocampus, similar to those levels found in young, 3-month-old, vehicle-treated mice [20]. In contrast, fluoxetine failed to increase mRNA expression of the majority of plasticity markers examined [20].

The purpose of the present study was to perform a retrospective cross-species network analysis to determine whether shared biological systems rather than single biomarkers are regulated in response to vortioxetine treatment. We explored whether common underlying biological mechanisms can be linked from distinct vortioxetine studies [20, 24]. A protein–protein interaction analysis is a stringent and effective method to uncover shared networks. We hypothesized that common networks (biological systems of interacting proteins) are engaged in rodent models in response to various vortioxetine treatment regimens. The network analysis was performed with qPCR data for selected sets of biomarkers in two distinct models (with little overlap in the two biomarker sets), and thus full-scale overrepresentation analyses were not possible. The limited nature of the qPCR datasets inevitably introduces a bias to the study and forces limitations to which parts of the protein interactome can be queried for regulated networks. With the lack of global data, we therefore employed alternative approaches to discover the protein interaction networks, including the investigated biomarkers that might drive the response to vortioxetine in two distinct animal models differing in species, sexes, brain regions, and treatment regimens.

## Methods

### Animals

All animal procedures were in accordance with Lundbeck Research U.S.A. Institutional Animal Care and Use Committee and NIH federal guidelines. Adult male Sprague–Dawley rats (8–12 weeks) and middle-aged female C57BL/6 mice (11 months) were obtained from Charles River (Wilmington, MA, USA) and were kept in a 12:12 light:dark cycle with ad libitum access to food and water.

### Dosing and RNA isolation

A summary of studies and experimental paradigms is shown in Table 1. For acute studies, adult male Sprague–Dawley rats received vehicle or vortioxetine (generated by H. Lundbeck A/S, Valby, Denmark) (10 mg/kg, i.p.), a clinically-relevant dose corresponding to full rSERT occupancy and ~80% occupancy at the r5-HT_1B_ receptor, at 2, 8, 12, or 27 h prior to harvesting the frontal cortex. RNA purification, cDNA synthesis, and quantitative real-time polymerase chain reaction (qPCR) using SYBR green were carried out as described elsewhere [24, 25]. Transcript levels of 80 genes involved in serotonergic and glutamatergic neurotransmission as well as neuroplasticity were assessed.

**Table 1:** Summary of studies and experimental paradigms

Study	Species	Sex	Age (weeks)	Dosing Regimen	Brain Regions	Figure(s)	References
Network analysis	Mouse	Female	48	Chronic	Hippocampus	Figures 2, 3, 4, 5 and Additional file 1: Table S1	Li et al. [20]
Network analysis	Rat	Male	8	Acute	Frontal cortex	Figures 2, 3, 4, 5 and Additional file 1: Table S2	du Jardin et al. [24]
OpenArray qPCR	Rat	Male	12	Chronic	Frontal cortex, Hippocampus	Figure 6	Waller-et al. [27]

Outline of experimental conditions for mouse and rat studies used in the network analysis and for the OpenArray qPCR study for biological validation of the network analysis. See references for additional details regarding these studies including animal models and differentiation to other antidepressants

For chronic studies in mouse, aged female C57BL mice received 1 month treatment of vehicle or vortioxetine (H. Lundbeck A/S) (0.6 g base per kg food) in Purina 5001 rodent chow (Research Diets Inc., New Brunswick, NJ), a clinically-relevant dose that fully occupies the mSERT in addition to ~50% occupancy at the m5-HT_1B_ receptor [20]. mRNA levels of several neuroplasticity genes were measured in the hippocampus using qPCR as previously described [20].

For subsequent chronic vortioxetine studies to validate the network analysis, adult male Sprague–Dawley rats, *n* = 12/group, received 0.22 g/kg of vortioxetine (H. Lundbeck A/S) food (Research Diets Inc.) (corresponding to ~50% rSERT occupancy), 0.6 g/kg vortioxetine food (Research Diets Inc.) (corresponding to full rSERT occupancy and ~50% occupancy at r5-HT_1B_), or 1.8 g/kg vortioxetine food (Research Diets Inc.) (achieving full occupancy at rSERT and ~90% occupancy at r5-HT_1B_) for 1 month (also see [28]). Gene expression was assessed in the frontal cortex and hippocampus.

Cortex and hippocampus were rapidly dissected and stored in RNAlater (Ambion; Life Technologies, Carlsbad, CA, USA) at −20 °C prior to processing. Tissue was homogenized on ice in 1 ml of lysis buffer (Ambion RNAqueous 96 kit) using an Autogizer (Tomtec, Hamden, CT, USA). Total RNA was extracted from an aliquot of the lysate using the Ambion RNAqueous 96 automated kit according to the manufacturer’s protocol. Following RNA elution from the column, a second DNase digestion was added to eliminate any residual genomic DNA in the samples. The total RNA was evaluated with an Agilent Bioanalyzer 2100 to determine RNA concentration and integrity. The average RNA integrity number (RIN) values were 6.7 for the cortex and 6.3 for the hippocampus. RNA concentration was normalized to 20 ng/µl and reverse transcription was performed using 200 ng of RNA and Superscript VILO (Life Technologies, Carlsbad, CA, USA) according to the manufacturer’s protocol. The Quant-It dye intercalation assay (Life Technologies, Carlsbad, CA, USA) was used to determine cDNA yield, and the samples were normalized to a concentration of 3 ng/µl. mRNA levels of various plasticity-related genes and receptors were examined by OpenArray qPCR, described below.

### Network and protein–protein interaction analysis

Gene expression analysis and integrative modeling for network identification were performed by Intomics A/S (Lyngby, Denmark). The main source of network data used in the analysis was the experimentally derived physical protein–protein interaction database InWeb_IM [26]. Briefly, InWeb_IM is a large, robust, high confidence database of inferred human physical protein–protein interactions gathered from multiple databases of experimental evidence. For a full description of the resource including background databases and scoring algorithms, please refer to [26]. This database has been shown to have a very high coverage of interaction data for brain-regulated genes, suggesting it to be particularly useful for discovery of new pathway relationships in neuropsychiatric diseases [26]. Two different types of protein-interaction networks were derived from the InWeb_IM database (February 2014 version):

1. Networks comprised of transcriptionally-regulated proteins and their interaction partners.
2. Pre-defined protein interaction networks from the Intomics in-house protein Complex Catalogue of 2412 topological clusters found in the full network space, built to facilitate analyses based on fixed networks. The complex catalog was generated by considering all 1st order networks around receptors and signaling pathway-related proteins annotated in UniProt and KeGG, pruning the networks for “sticky proteins” (proteins with a high number of interactions outside the current network), and then merging all networks with a high degree of overlap (>95%).

The protein interaction networks can be described as *draft pathways* as they are typically not as well-described as literature-derived canonical pathways. They however hold a valuable potential for discovery of new insight, as the use of protein–protein interaction data allows for an analysis that goes beyond what is currently annotated in literature-derived pathways.

To facilitate the network analysis, mouse and rat genes were mapped to human orthologs using the multiple orthology database approach outlined in [26], to ensure correct mapping in cases of uncertainty, and qPCR values were log_2_ transformed. Subsequently, quality control plots of the log_2_-transformed values were generated to identify outliers. The Mann–Whitney U test was used to calculate *p* values, which were then adjusted using the Benjamini–Hochberg procedure for correction for multiple testing.

**Fig. 1:**
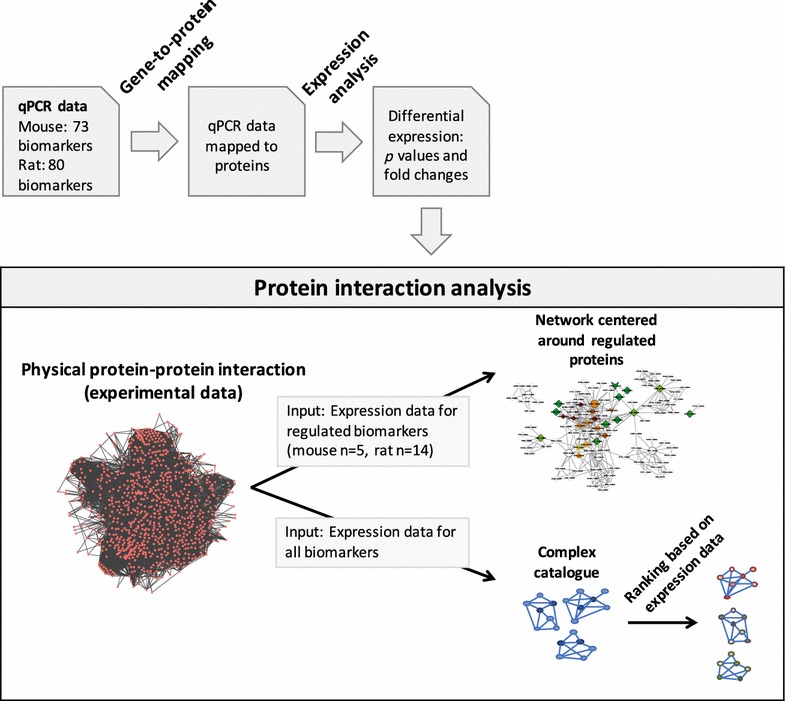
Schematic of procedure for network analysis based on differentially-regulated targets. Work-flow for generating protein networks. Following mapping of targets to human orthologues and differential expression analysis of qPCR data, significantly-regulated targets were used to build networks of protein–protein interactions

The following approaches were used for identification of networks significantly associated with the gene expression data (see Fig. 1 for an overview of the workflow):

1. Genes found to be significantly regulated in the gene expression analysis were used to seed a “virtual pulldown” in the full experimentally-derived, protein–protein interaction database: Other proteins directly interacting with the protein products of the regulated genes were found and added to the network based on their degree of interconnectivity, and interactions between those proteins were reported as well. Since these protein interaction clusters from the human interactome are based on experimental data and encompass a large range of proteins, this approach has the potential to extract new information about the underlying biology affected by transcriptional regulation.
2. The *p* values from all tested genes were mapped onto the corresponding proteins in the Complex Catalogue, and for each network, a combined score was calculated using Fisher’s combined probability test. The 839 networks that contained any of the measured biomarkers were ranked according to this combined score, and the best scoring networks were investigated.

### OpenArray qPCR platform

OpenArray qPCR was performed as described previously [20, 27]. Briefly, pre-amplification of the samples was accomplished using 12 cycles of PCR in a reaction containing 10 ng of cDNA, 112 primer sets exactly matching targets on the OpenArray chip, and 2× Taqman Preamp master mix (Life Technologies, Carlsbad, CA, USA) according to the vendor’s protocol. A complete list of targets tested can be found in Additional file 1: Table S3. Amplified samples were analyzed on a QuantStudio 12K flex instrument (Life Technologies, Carlsbad, CA, USA). Data analysis was performed using the Expression Suite software package provided with the instrumentation. Global normalization across the entire chip was used to adjust the raw expression values for all targets. More specifically, within each sample, the geometric mean of the comparative cycle threshold (Ct) values for all of the qPCR assays was calculated. Using this value, a delta Ct was calculated for every assay in each sample (geometric mean for sample X minus the Ct for assay 1, assay 2, etc., across the sample). These delta Ct values were then used to calculate expression levels in the control and different treatment groups, which then enabled differential expression levels to be determined. The reported relative expression (RQ) value represents the ratio of normalized expression in each treatment group divided by the normalized expression in the control group. Values are denoted as mean ± SEM. Statistical significance is defined as a false discovery rate (FDR) *p* value of *p* < 0.05.

## Results

### Network analysis reveals common biological systems related to plasticity and development are modulated in response to vortioxetine in mouse and rat

Previous qPCR studies examining expression of various neuroplasticity-related genes in adult rats treated with acute vortioxetine and middle-aged mice treated with chronic vortioxetine revealed upregulation of various signal transduction, plasticity, and neurotransmission-related genes [20, 24]. We sought to determine whether there is overlap and common biological networks regulated in response to vortioxetine, as variation is typically evident at the network level and there was little overlap in the markers examined in these two qPCR studies. Following comprehensive mapping to orthologous human proteins and differential expression analysis, including correction for multiple testing (Additional file 1: Tables S1 and S2), differentially-regulated targets were used to generate networks, as described above. Comparisons between vehicle and vortioxetine treatments in middle-aged mice (*n* = 10 animals for vehicle and vortioxetine) and adult rat samples (*n* = 5 animals for vehicle, and *n* = 6 animals for vortioxetine) were used for the differential expression analysis. Following corrections for multiple testing, 5 mouse genes related to plasticity, *Arc*, *Fmr1*, *Ndor1*, *Shank1*, and *Slc6a3*, were significantly regulated (adjusted *p* value of <0.05 (Table 2). There were no significant genes after the rat targets were corrected for multiple testing, most likely due to the lower *n* values in the rat study. Thus, the differentially-regulated targets before correction were used for the network analysis. A total of 14 plasticity-related genes including, *Cacng2*, *Cacng6*, *Cacng8*, *Grik4*, *Grik5*, *Grm1*, *Grm5*, *Grm7*, *Homer3*, *Htr1b*, *Mtor*, *Ppp1r9b*, *Prkca*, and *Syn3*, were significantly upregulated following differential analysis (Table 3).

**Table 2:** Biomarkers significantly upregulated by vortioxetine in mouse hippocampus (Old VEH vs. Old VOR)

Gene name	Protein ID	BH^a^ corrected *p* value
*Arc*	ARC	0.0174
*Fmr1*	FMR1	0.0217
*Ndor1*	NDOR1	0.0174
*Shank1*	SHANK1	0.0217
*Slc6a3*	SC6A3	0.0174

Significance is determined relative to vehicle (VEH) treatment (*n* = 10 animals per group). Following correction for multiple testing, 5 genes were significantly upregulated and used as inputs for the subsequent network analysis

^a^Benjamini–Hochberg correction for multiple testing

**Table 3:** Biomarkers significantly upregulated by vortioxetine in rat frontal cortex (VEH vs. VOR)

Gene name	Protein ID	Un-corrected *p* value <0.05
*Cacng2*	CCG2	0.0173
*Cacng6*	CCG6	0.0173
*Cacng8*	CCG8	0.0173
*Grik4*	GRIK4	0.0095
*Grik5*	GRIK5	0.0095
*Grm1*	GRM1	0.0095
*Grm5*	GRM5	0.0095
*Grm7*	GRM7	0.0095
*Homer3*	HOMER3	0.0095
*Htr1b*	5-HT1B	0.0173
*Mtor*	MTOR	0.0317
*Ppp1r9b*	Spinophilin/NEB2	0.0303
*Prkca*	KPCA	0.0303
*Syn3*	SYN3	0.0317

Significance is determined relative to VEH (saline) treatment (*n* = 5 animals for VEH; *n* = 6 animals for VOR). Due to the lower sample size, significance was not achieved following correction for multiple testing and thus, significant targets before correction were determined. A total of 14 targets were significantly modulated in response to VOR treatment and used as inputs in the network analysis

**Fig. 2:**
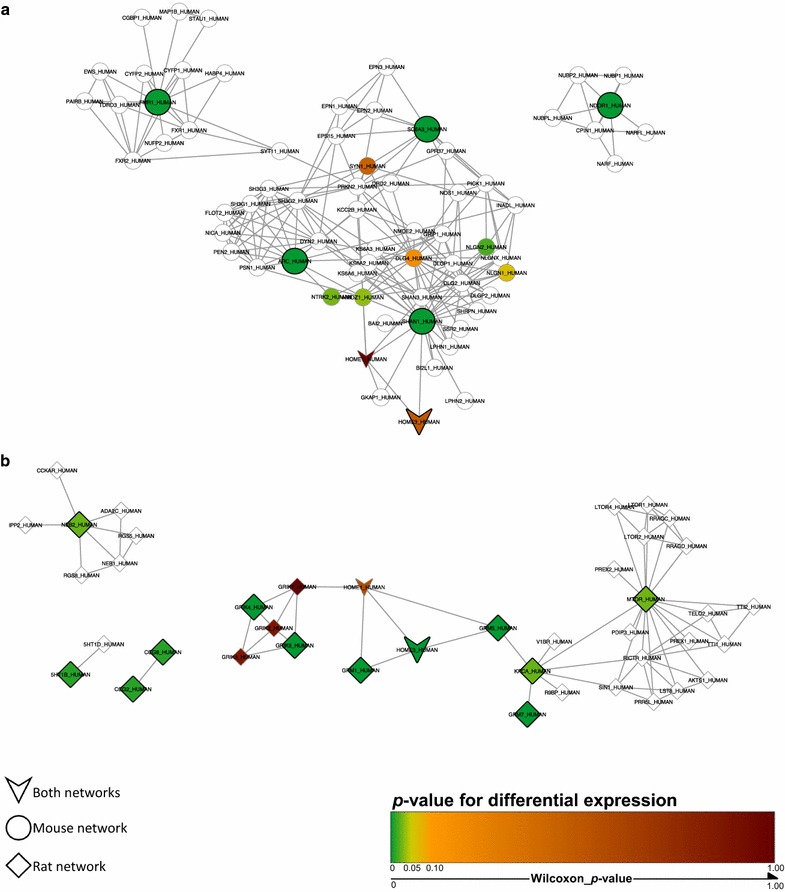
Mouse and rat networks mapped to human proteins. **a** Mouse network. The 5 significantly-regulated targets following correction for multiple testing were used to generate a protein–protein interaction network. The genes highlighted in *green* represent the 5 differentially-regulated mouse genes. *p* = 0.001652, Wilcoxon, enrichment of significantly-regulated biomarkers relative to vehicle-treated mice. **b** Rat network. The 14 differentially-regulated genes before correction for multiple testing were used for the network analysis. The genes highlighted in *green* represent the 14 targets that showed significance following differential analysis. *p* = 0.00000642, Wilcoxon, enrichment of significantly-regulated targets in comparison to vehicle-treated rats. The *scale* indicates *p* values for significance of differential expression, ranging from *green* (*p* = 0) to *dark red* (*p* = 1)

**Fig. 3:**
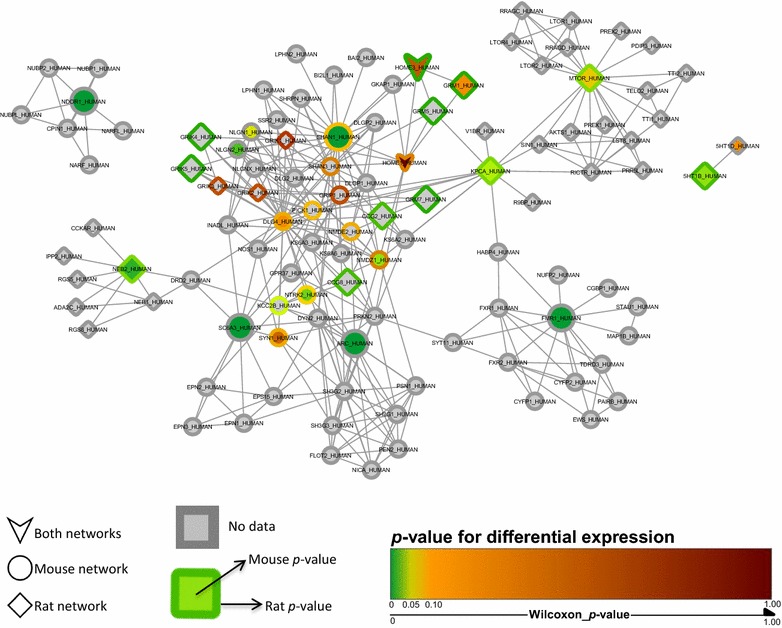
Merged mouse and rat network (mapped to human proteins). The colored genes were used to build the analysis/network of protein–protein interaction partners. The *squares with colored borders* represent targets from the rat network, and *colored circles* indicate targets from the mouse network. The *arrowheads* indicate the common targets found in mouse and rat networks. *p* = 0.0000808, Wilcoxon, mouse data, enrichment of regulated biomarkers. *p* = 0.00000217, Wilcoxon, rat data, enrichment of regulated biomarkers. The scale denotes *p* values for significance of differential expression, ranging from *green* (*p* = 0) to *dark red* (*p* = 1). Targets with *colored borders* represent the rat *p* value, and *center colors* indicate mouse *p* values. *Gray* represents targets in which no qPCR data were obtained

**Fig. 4:**
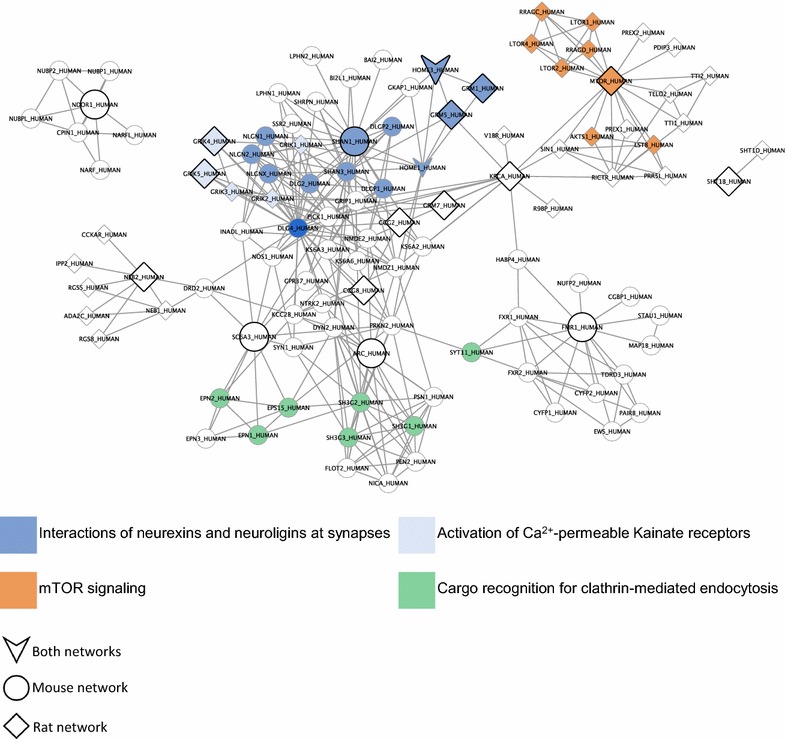
Merged mouse and rat network (mapped to human proteins) and overlapping Reactome pathways. Genes are colored according to their Reactome pathway memberships (DLG4 is part of both ‘Interactions of neurexins and neuroligins at synapses’ and ‘Activation of Ca^2+^-permeable Kainate receptors’ pathways). The most prevalent Reactome pathways are shown. The network includes 13 (equal to 22%) of the proteins from the ‘Interactions of neurexins and neuroligins at synapses’ pathway, 6 (equal to 60%) of the proteins from the ‘Activation of Ca^2+^-permeable Kainate receptors’ pathway, 8 (equal to 50%) of the proteins from the ‘mTOR signaling’ pathway, and 7 (equal to 7%) of the proteins from the ‘Cargo recognition for clathrin-mediated endocytosis’ pathway. Reactome pathway annotations were filtered to include only reviewed human proteins from UniProt and protein-pathway associations with the evidence code “traceable author statement” (TAS)

Subsequently, these differentially-regulated genes were used as seeds to query the InWeb_IM database of interacting proteins, and the 1st order protein–protein networks shown in Fig. 2 were identified. The mouse and rat networks only had two proteins in common (Homer protein homolog 1 and Homer protein homolog 3), but overlaying the networks revealed that the other protein members of the two networks were interconnected. Furthermore, merging the two networks yielded a single network capable of explaining the transcriptional response in both model organisms (Fig. 3). This biological network of 109 proteins contained clusters of interacting proteins related to neuronal development, including Notch signaling and neuron formation and outgrowth, synaptic transmission, receptor and mTOR signaling, synaptic plasticity, metabolism, and cell growth and apoptosis, and was significantly engaged in both mouse and rat (Additional file 2: Figure S1). For each protein in the network, membership of Reactome pathways was established using the official Reactome mapping file (UniProt2Reactome.txt, downloaded from www.reactome.org, December 21, 2016). The mapping file was processed to only include reviewed human proteins from UniProt and protein-pathway associations with the evidence code “traceable author statement” (TAS). The most prevalent Reactome pathways with respect to overlap in proteins with the merged mouse and rat network were *Interactions of neurexins and neuroligins at synapses*, *Activation of Ca* ^*2*+^-*permeable Kainate receptors*, *mTOR signaling,* and *Cargo recognition for clathrin*-*mediated endocytosis*. The protein overlap with these pathways is shown in Fig. 4. Applying the Wilcoxon rank sum test to mouse and rat data, respectively, revealed that *p* values for differential expression were significantly lower for biomarkers within the network than for other tested biomarkers in both species [mouse: Wilcoxon rank sum test *p* = 0.0000808 for enrichment of low *p* values for biomarkers in the network (*n* = 19) compared to the remaining measured biomarkers (*n* = 53); rat: Wilcoxon rank sum test *p* = 0.00000217 for enrichment of low *p* values for biomarkers in the network (*n* = 28) compared to the remaining measured biomarkers (*n* = 52)], indicating a shared biology in response to vortioxetine in two distinct model systems examining a different set of biomarkers. Even with the low number of genes examined in both datasets, meaningful biological data could be extracted using this method to elucidate potential molecular mechanisms underlying vortioxetine treatment.

### Protein complex analysis confirms neuroplasticity and development-related biomarkers are regulated by vortioxetine in mouse and rat

**Fig. 5:**
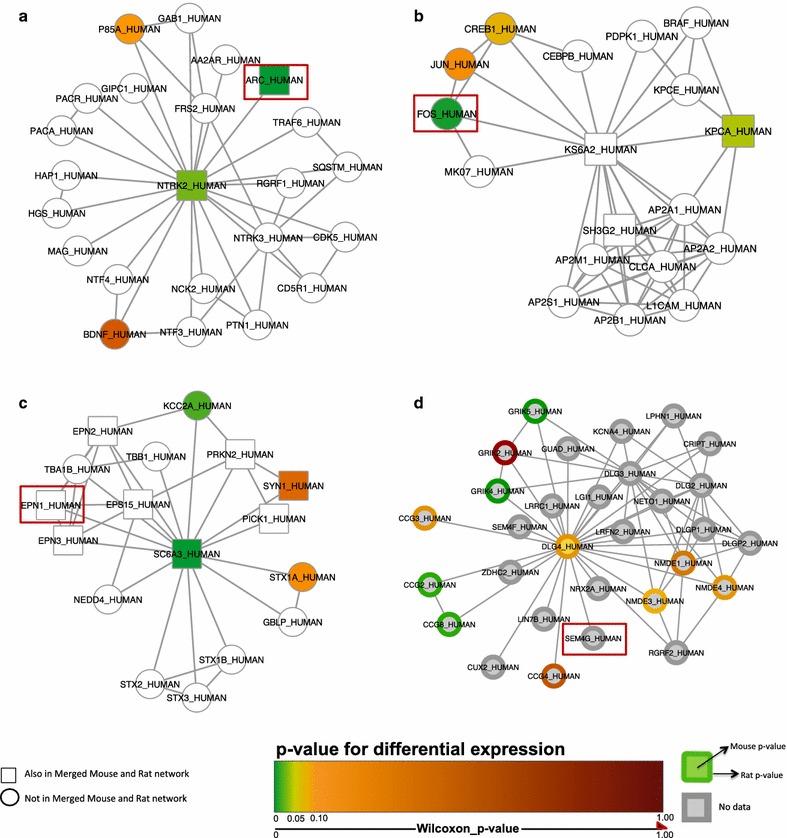
Protein complex analysis. **a** Complex 1544—the Arc pathway, synaptic plasticity-related, is regulated by vortioxetine treatment at the mRNA level. **b** Complex 85—transcriptional regulation, including FOS modulated by vortioxetine at the mRNA level. **c** Complex 163—endocytosis/neurotransmitter release pathways, including EPN1 that was upregulated in the qPCR study. **d** DLG4/PSD-95 network, including SEMA4G that was upregulated in the qPCR study. The *connecting lines* represent physical protein–protein interactions. The targets outlined in *red* indicate genes modulated in response to chronic vortioxetine treatment in adult rats. The *scale* indicates *p* values for significance of differential expression, ranging from *green* (*p* = 0) to *dark red* (*p* = 1). Targets with *colored borders* represent the rat *p* value, and *center colors* indicate mouse *p* values. *Gray* represents targets in which no qPCR data were obtained

Ranking the fixed protein–protein interaction networks (draft pathways) from the Complex Catalogue of topological clusters of physically-interacting proteins based on their regulation in the qPCR data led to the identification of networks significantly associated with the vortioxetine response (Fig. 5). Gene ontology overrepresentation analyses performed with a custom implementation of the GeneMerge algorithm [28] and the Gene Ontology Consortium GoSlim annotations (accession date May 14, 2014) revealed biological functions in the networks related to common themes of synaptic plasticity, transmission, and neurodevelopment. These networks included the neuronal activity marker ARC (Fig. 5a), transcriptional regulator FOS (Fig. 5b), EPN1 (Epsin 1), associated with endocytosis and actin remodeling (Fig. 5c), and SEMA4G (Semaphorin 4g), related to neurodevelopment and plasticity (Fig. 5d).

### qPCR analysis provides biological validation of targets identified in the network analysis

**Fig. 6:**
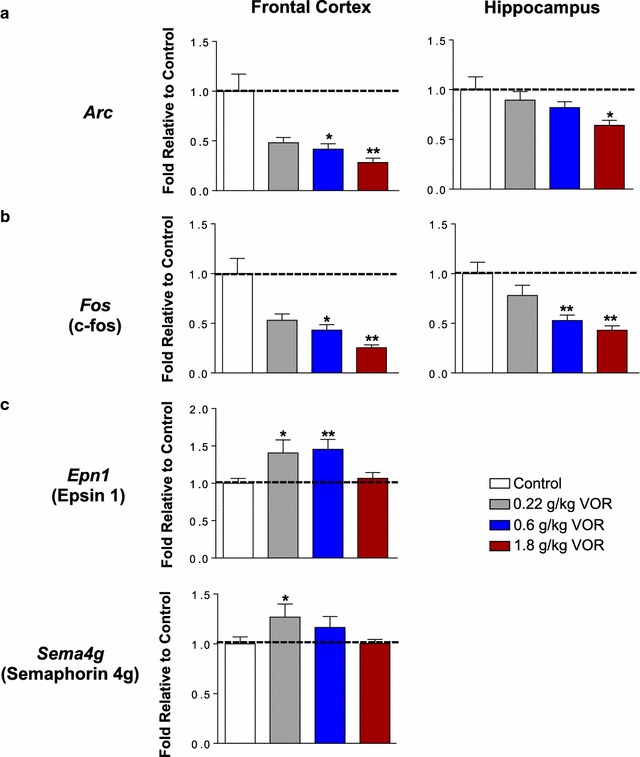
Vortioxetine significantly regulates biomarkers within the protein–protein interactome identified by the network analysis. qPCR analysis following chronic vortioxetine (VOR) administration at a range of doses reveals genes identified in the network analysis are significantly modulated by vortioxetine. **a** Plasticity-related targets. *Arc* is significantly downregulated in response to 0.6 and 1.8 g/kg VOR in the frontal cortex and in response to 1.8 g/kg VOR in the hippocampus. Cortex: *p = 0.021, 0.6 g/kg versus control (Ctrl); **p = 0.005, 1.8 g/kg versus Ctrl. Hippocampus: *p = 0.033, 1.8 g/kg versus Ctrl. **b** Transcription factors. *Fos* is significantly downregulated in response to 0.6 and 1.8 g/kg VOR in the frontal cortex and hippocampus. Cortex: *p = 0.034, 0.6 g/kg versus Ctrl; **p = 0.007, 1.8 g/kg versus Ctrl. Hippocampus: **p = 0.009, 0.6 g/kg versus Ctrl; **p = 0.004, 1.8 g/kg versus Ctrl. **c** Endocytosis/actin remodeling and neurodevelopment/plasticity. *Epn1* is significantly upregulated in response to 0.22 and 0.6 g/kg VOR treatment in the frontal cortex. *p = 0.070, 0.22 g/kg versus Ctrl; **p = 0.001, 0.6 g/kg versus Ctrl. *Sema4g* is significantly upregulated following 0.22 g/kg vortioxetine treatment in the frontal cortex. *p = 0.040, 0.22 g/kg versus Ctrl. All values are represented as fold change relative to the control group and denoted as mean ± SEM. Statistical significance is defined as a FDR *p* value of *p* < 0.05. *n* = 12 animals per group

The discovery of additional markers in the protein interaction space not evaluated in the original qPCR studies led us to investigate whether these markers are also modulated by vortioxetine. Thus, we performed a follow-up qPCR study as biological validation of the network analysis. Adult rats received chronic vortioxetine at a range of doses with varying SERT and 5-HT receptor occupancies, and mRNA levels of various plasticity markers and receptors were measured in the frontal cortex and hippocampus and compared to the control treatment group. We found that the same biology was regulated in this model system. *Arc*, involved in synaptic and structural plasticity, was downregulated in response to 0.6 g/kg (0.42 ± 0.05, VOR vs. 1.00 ± 0.17, Ctrl) and 1.8 g/kg (0.28 ± 0.04, VOR, vs. 1.00 ± 0.17, Ctrl) vortioxetine in the frontal cortex and in response to 1.8 g/kg (0.64 ± 0.05, VOR vs. 1.00 ± 0.13, Ctrl) vortioxetine in the hippocampus relative to control (Fig. 6a) (also see [27]). The transcription factor *Fos* was also downregulated following chronic 0.6 g/kg (Cortex: 0.43 ± 0.06, VOR vs. 1.00 ± 0.15, Ctrl; Hippocampus: 0.53 ± 0.06, VOR vs. 1.00 ± 0.12, Ctrl) and 1.8 g/kg (Cortex: 0.25 ± 0.03, VOR vs. 1.00 ± 0.15, Ctrl; Hippocampus: 0.43 ± 0.05, VOR vs. 1.00 ± 0.12, Ctrl) vortioxetine treatment in the frontal cortex and hippocampus (Fig. 6b) (also see [27]). Of the additional biomarkers identified by the network analysis, *Epn1* (Epsin 1), associated with endocytic functions and actin remodeling, was upregulated in the frontal cortex by 0.22 g/kg (1.41 ± 0.18, VOR vs. 1.00 ± 0.06, Ctrl) and 0.6 g/kg (1.46 ± 0.13, VOR vs. 1.00 ± 0.06, Ctrl) vortioxetine treatment, and the neurodevelopmental and plasticity biomarker *Sema4g* (Semaphorin 4g) was also upregulated by 0.22 g/kg (1.27 ± 0.13, VOR vs. 1.00 ± 0.07, Ctrl) vortioxetine treatment in the frontal cortex (Fig. 6c) (also see [27]).

## Discussion

We have performed a retrospective cross-species network analysis of distinct datasets and studies to determine whether common biological systems are modulated in response to vortioxetine treatment. We found significant overlap between mouse and rat and reveal that vortioxetine regulates shared biological networks in two distinct animal models differing in species, sexes, brain regions, and treatment regimens. Moreover, a subsequent additional qPCR analysis revealed that the same biology is modulated in response to chronic vortioxetine treatment of adult rats. This indicates that common biological mechanisms are regulated in response to vortioxetine treatment.

The mRNA levels of targets modulated by vortioxetine treatment provide a link to the role of vortioxetine in improved pro-cognitive performance in preclinical and clinical studies. The immediate early genes (IEGs) ARC and FOS, markers of neuronal activation, are consistently modulated in response to vortioxetine treatment. ARC is a cytoskeletal protein involved in various forms of synaptic plasticity, including LTP, long-term depression, and homeostatic plasticity, as well as morphological plasticity [29, 30]. Given the role of vortioxetine in enhancing LTP in vitro in hippocampal slices [13] and increasing dendritic branching and dendritic spine density in vivo [14, 15], regulation of ARC by vortioxetine may contribute to these effects on synaptic and structural plasticity. The IEG FOS is also induced by neuronal activity and plays a role in induction of NMDA receptor-dependent LTP and in hippocampus-dependent spatial learning tasks [31]. Regulation of FOS expression by vortioxetine in the hippocampus may also contribute to its effect on enhancing LTP and visuospatial learning in middle-aged mice [20].

The transcription of two biomarkers detected in the network analysis, EPN1 (Epsin 1) and SEMA4G (Semaphorin 4g) was confirmed by qPCR analysis to be upregulated by vortioxetine treatment (also see [27]). The adaptor protein EPN1 interacts with neurotransmitter re-uptake machinery in the presynaptic region and is involved in clathrin-mediated endocytosis and vesicle budding, both of which are actin-dependent events [32]. It also plays a role in transcriptional regulation and mediates actin polymerization and assembly [33–36]. Regulation of EPN1 expression by vortioxetine may influence its role in actin remodeling and in maintenance of dendritic spine structure [16]. Semaphorins have predominant roles in axon guidance and neurodevelopment, but are continually expressed into adulthood where they can mediate synapse stability and hippocampal plasticity [37, 38]. SEMA4G interacts with the major postsynaptic scaffold protein postsynaptic density 95 (PSD-95) [38], which links the NMDA receptor complex to downstream signaling pathways. Thus, SEMA4G may function in refining postsynaptic specializations and modulating synaptogenesis and NMDA-mediated signal transduction pathways, processes relevant to vortioxetine’s role in plasticity and cognitive-enhancing performance.

The differential regulation of specific targets in mouse versus rat may be partly due to treatment-specific effects. Chronic vortioxetine was administered in middle-aged mice, which predominantly showed modulation of IEG expression, whereas adult rats, in which calcium channel and glutamate receptor expression was significantly regulated, were treated with acute vortioxetine. In line with these findings, chronic vortioxetine treatment in adult rats can promote cell-type specific regulation of c-fos protein expression in certain subregions of the hippocampus (ongoing study, yet unpublished). Accordingly, cell-type specific effects in response to vortioxetine treatment may be diluted in whole tissue preparations, and expression of these targets at the protein level may be regulated differently. However, the distinct targets in mouse versus rat likely converge on common downstream pathways. Glutamate receptor and calcium channel activity can modulate IEG expression as well as transcription of synaptic targets. Stimulation of metabotropic glutamate receptors and subsequent elevated calcium responses lead to an increase in dendritic Arc levels, and this increase is abrogated by calcium channel blockers [39]. NMDAR hypofunction in mice deficient in serine racemase (SR), the enzyme that converts l-serine to the NMDAR coagonist d-serine, leads to reduced Arc expression in the hippocampus [40]. Conversely, acute d-cycloserine treatment to partially activate NMDARs at a dose corresponding to enhanced memory acquisition and consolidation promotes increased Arc protein levels in the hippocampus [41]. In addition, calcium influx through voltage-sensitive calcium channels triggers CREB-mediated transcription of c-fos [42, 43]. Thus, the differentially-regulated targets in mouse versus rat may ultimately impact similar plasticity mechanisms.

Although sex-specific differences prevail in MDD and the antidepressant response [44–48], we find similar biological networks modulated in female mice and male rats following chronic and acute vortioxetine treatment, respectively. Neurosteroids induce profound effects on neuronal activity and memory. Estradiol augments LTP, elevates dendritic spine density in the hippocampus, and enhances cognitive performance in hippocampal-dependent memory tasks [49–51]. Moreover, progesterone metabolites target the inhibitory GABA_A_ receptor and can therefore influence cognitive function [52]. In addition, androgens maintain hippocampal-dependent plasticity and cognitive function [53]. Testosterone-depleted rats exhibit elevated hippocampal CA3 mossy fiber LTP and branching [54], which may be compensating for decreased CA1 spine synapses [53]. At the behavioral level, endogenous testosterone in the prefrontal cortex has been correlated with enhanced working and reference memory during spatial learning [55]. Furthermore, testosterone-derived steroids can have neuroprotective effects [56–58]. Neurosteroids can also act as positive modulators of NMDA receptors and increase NMDA receptor surface expression [59, 60]. Thus, analogous regulation of pathways in female and male rodents may be related to a certain extent to the effects of neurosteroids on synaptic plasticity and cognitive processes.

## Conclusions

A central goal has been to identify novel biomarkers that play a role in MDD and may predict treatment response in MDD. However, due to the heterogeneity of MDD and variations in response to different treatments across different patient populations, it is difficult to attribute a single gene as a predictor of treatment response. In addition, cognitive dysfunction is prevalent in MDD [61], and vortioxetine alleviates cognitive dysfunction in various preclinical models [17–21] as well as in clinical studies [62–64]. We show here that vortioxetine consistently modulates the transcription of genes in synaptic plasticity-related networks, and the same biological mechanisms are engaged in distinct model systems, providing support for the neuroplasticity hypothesis of depression. Thus, the pro-cognitive characteristics of vortioxetine may be achieved by enhancing signaling in these networks.

## Additional files

**Additional file 1: Table S1.** Differential expression analysis of mouse hippocampal qPCR data. A panel of 73 biomarkers was examined in a chronic vortioxetine study in aged mice and subject to differential analysis for identification of significantly-regulated targets to generate protein–protein interaction networks. **Table S2**. Differential expression analysis of rat frontal cortex qPCR data. A panel of 80 biomarkers was evaluated in an acute vortioxetine study in adult rats and subject to differential analysis for identification of differentially-regulated targets to build protein–protein interaction networks. **Table S3**. List of targets examined in OpenArray study. Gene expression levels of biomarkers related to transcriptional regulation, signal transduction, synaptic plasticity, neurotransmitter release, neurodevelopment, and degradation, and of receptors and channels were evaluated in a chronic vortioxetine study in adult rats. The targets outlined in red indicate additional genes identified in the network and pre-defined complex analyses.

**Additional file 2: Figure S1.** Merged mouse and rat network (mapped to human proteins) and summary of biological functions of each sub-network. Biological functions were manually extracted from the Function and Gene Ontology fields of the UniProt protein entries. The genes with dark, bold borders were used to build the network of protein–protein interaction partners. Squares with bold borders represent upregulated targets from the rat network, and circles with bold borders indicate differentially-regulated targets from the mouse network. The arrowheads indicate the common targets found in mouse and rat networks. This network of physically-interacting proteins containing clusters related to synaptic plasticity, synaptic transmission, neurodevelopment, cell growth, metabolism, and apoptosis, was significantly modulated in both mouse and rat.

## Abbreviations

MDD: major depressive disorder
AMPA: α-amino-3-hydroxy-5-methyl-4-isoxazolepropionic acid
NMDA: *N*-methyl-d-aspartate
5-HT: serotonin
VOR: vortioxetine
SERT: 5-HT transporter
LTP: long-term potentiation
SSRI: selective serotonin-reuptake inhibitor
Ct: comparative cycle threshold
RQ: relative expression
FDR: false discovery rate
EPN1: Epsin 1
SEMA4G: Semaphorin 4g
IEG: immediate early gene
PSD-95: postsynaptic density 95
