# Supplementary material for: Neuroplasticity pathways and protein-interaction networks are modulated by vortioxetine in rodents
Source: BMC Neurosci. 2017 Aug 4;18:56. doi: 10.1186/s12868-017-0376-x (PMC5543755; doi:10.1186/s12868-017-0376-x)
Supplement: Supplementary file 1 — Additional file 1: Table S1. Differential expression analysis of mouse hippocampal qPCR data. A panel of 73 biomarkers was examined in a chronic vortioxetine study in aged mice and subject to differential analysis for identification of significantly-regulated targets to generate protein–protein interaction networks. Table S2. Differential expression analysis of rat frontal cortex qPCR data. A panel of 80 biomarkers was evaluated in an acute vortioxetine study in adult rats and subject to differential analysis for identification of differentially-regulated targets to build protein–protein interaction networks. Table S3. List of targets examined in OpenArray study. Gene expression levels of biomarkers related to transcriptional regulation, signal transduction, synaptic plasticity, neurotransmitter release, neurodevelopment, and degradation, and of receptors and channels were evaluated in a chronic vortioxetine study in adult rats. The targets outlined in red indicate additional genes identified in the network and pre-defined complex analyses. [file 12868_2017_376_MOESM1_ESM.pdf]

Mouse protein ID	Human protein ID	case mean expression	control mean expression	Mean log2 fold change	case median expression	control median expression	Median log2 fold change	Wilcoxon p-value	Wilcoxon test statistic	BH-corrected p-value
NDOR1_MOUSE	NDOR1_HUMAN	6.875285818	6.635259899	0.240025919	6.894077423	6.66163	0.232447423	0.000324753	94	0.017406742
SC6A3_MOUSE	SC6A3_HUMAN	7.1449316	6.633644044	0.511287556	6.984185521	6.605138629	0.379046893	0.000725281	92	0.017406742
ARC_MOUSE	ARC_HUMAN	7.073697975	6.618851066	0.454846909	7.0865718	6.579997144	0.506574656	0.000725281	92	0.017406742
FMR1_MOUSE	FMR1_HUMAN	6.939980518	6.634371373	0.305609145	6.908806673	6.622417714	0.286388958	0.001504687	90	0.021667497
SHAN1_MOUSE	SHAN1_HUMAN	6.815832494	6.641022945	0.174809549	6.818261158	6.660088192	0.158172967	0.001504687	90	0.021667497
FOS_MOUSE	FOS_HUMAN	7.239764828	6.550835027	0.688929802	7.157572205	6.487438093	0.670134112	0.005196042	86	0.062352508
RAB3A_MOUSE	RAB3A_HUMAN	6.793301322	6.632241242	0.16106008	6.788279427	6.642401208	0.145878219	0.008930698	84	0.091858606
NFKB1_MOUSE	NFKB1_HUMAN	6.835221016	6.634394306	0.200826709	6.854684659	6.565811782	0.288872877	0.011496244	83	0.09196995
NEB2_MOUSE	NEB2_HUMAN	6.782259942	6.636224408	0.146035534	6.719653484	6.605589864	0.11406362	0.011496244	83	0.09196995
NLGN2_MOUSE	NLGN2_HUMAN	6.856260974	6.627501617	0.228759357	6.877983725	6.704790841	0.173192883	0.018543376	81	0.121374826
KCC2A_MOUSE	KCC2A_HUMAN	6.844311721	6.634219891	0.21009183	6.818699761	6.642510054	0.176189706	0.018543376	81	0.121374826
NTRK2_MOUSE	NTRK2_HUMAN	6.767165319	6.63905928	0.12810604	6.739035147	6.603772971	0.135262176	0.02880556	79	0.170222347
5HT1B_MOUSE	5HT1B_HUMAN	6.777753286	6.637479069	0.140274216	6.77175353	6.59710373	0.1746498	0.035462989	78	0.170222347
SCG2_MOUSE	SCG2_HUMAN	6.484468818	6.635106004	-0.150637187	6.463250595	6.648774555	-0.18552396	0.035462989	22	0.170222347
NMDZ1_MOUSE	NMDZ1_HUMAN	6.742953558	6.637119971	0.105833588	6.76638025	6.648105357	0.118274893	0.035462989	78	0.170222347
KPCA_MOUSE	KPCA_HUMAN	6.765608827	6.635209821	0.130399006	6.786312221	6.64946087	0.136851351	0.043257053	77	0.194656736
CBPE_MOUSE	CBPE_HUMAN	6.822015395	6.622105159	0.199910236	6.82982849	6.684678329	0.14515016	0.052425902	76	0.209703609
KAPCA_MOUSE	KAPCA_HUMAN	6.753913669	6.63401571	0.119897959	6.771285587	6.624576923	0.146708664	0.052425902	76	0.209703609
NLGN1_MOUSE	NLGN1_HUMAN	6.790050926	6.627113672	0.162937254	6.78463351	6.581119293	0.203514218	0.063012839	75	0.238785493
CAPS1_MOUSE	CAPS1_HUMAN	6.756708963	6.635594833	0.121114129	6.784895444	6.612103611	0.172791832	0.075256013	74	0.246292407
CREB1_MOUSE	CREB1_HUMAN	6.750673643	6.637233207	0.113440436	6.74174927	6.626803833	0.114945438	0.075256013	74	0.246292407
MTOR_MOUSE	MTOR_HUMAN	6.748077699	6.636577777	0.111499922	6.746155089	6.651455723	0.094699366	0.075256013	74	0.246292407
S100B_MOUSE	S100B_HUMAN	6.756023034	6.63513357	0.120889464	6.786604322	6.659272987	0.127331336	0.089209552	73	0.279264685
P85A_MOUSE	P85A_HUMAN	6.770246883	6.63321616	0.137030724	6.793588345	6.638806341	0.154782004	0.105122432	72	0.315367295
TF65_MOUSE	TF65_HUMAN	6.779321534	6.632961938	0.146359596	6.73751688	6.620168219	0.11734866	0.123005477	71	0.328014607
SYT9_MOUSE	SYT9_HUMAN	6.803852954	6.612462005	0.191390948	6.746719125	6.612635657	0.134083468	0.123005477	71	0.328014607
MECP2_MOUSE	MECP2_HUMAN	6.712005801	6.639048024	0.072957777	6.721335705	6.665532642	0.055803063	0.123005477	71	0.328014607
5HT7R_MOUSE	5HT7R_HUMAN	6.772261007	6.634912935	0.137348072	6.793233231	6.573417538	0.219815693	0.143140142	70	0.340444695
STXB1_MOUSE	STXB1_HUMAN	6.738388594	6.633398377	0.104990216	6.755211281	6.624987213	0.130224068	0.143140142	70	0.340444695
DLG4_MOUSE	DLG4_HUMAN	6.749224111	6.63858415	0.110639961	6.74647096	6.645006075	0.101464886	0.165493949	69	0.340444695
5HT1D_MOUSE	5HT1D_HUMAN	6.925629189	6.589745519	0.33588367	6.910187019	6.683475112	0.226711907	0.165493949	69	0.340444695
5HT5A_MOUSE	5HT5A_HUMAN	6.704471449	6.634387477	0.070083973	6.75949266	6.650630383	0.108862277	0.165493949	69	0.340444695
STX1A_MOUSE	STX1A_HUMAN	6.757227391	6.632014635	0.125212756	6.747790081	6.666807589	0.080982492	0.165493949	69	0.340444695
JUN_MOUSE	JUN_HUMAN	6.840056451	6.629239694	0.210816757	6.813132863	6.626421519	0.186711343	0.165493949	69	0.340444695
GRM1_MOUSE	GRM1_HUMAN	6.74155091	6.627264521	0.114286388	6.765134201	6.621126912	0.144007289	0.165493949	69	0.340444695
CMGA_MOUSE	CMGA_HUMAN	6.482123725	6.607402767	-0.125279042	6.458948702	6.522599427	-0.063650725	0.190315876	32	0.351352387
5HT3A_MOUSE	5HT3A_HUMAN	6.742448965	6.633838187	0.108610778	6.794613696	6.600276023	0.194337673	0.190315876	68	0.351352387
JAK2_MOUSE	JAK2_HUMAN	6.784249847	6.622285717	0.16196413	6.773359003	6.726125769	0.047233234	0.190315876	68	0.351352387
SYPH_MOUSE	SYPH_HUMAN	6.750731087	6.633172155	0.117558932	6.778433964	6.717718455	0.060715509	0.190315876	68	0.351352387
5HT4R_MOUSE	5HT4R_HUMAN	6.735481816	6.637521537	0.097960279	6.729014216	6.629354964	0.099659252	0.217562623	67	0.382061192
SYNPO_MOUSE	SYNPO_HUMAN	6.70471351	6.636347953	0.068365557	6.746491508	6.685684002	0.060807505	0.217562623	67	0.382061192
SC6A4_MOUSE	SC6A4_HUMAN	6.618547624	6.382413003	0.236134622	6.697063692	6.117187803	0.57987589	0.247450692	66	0.424201186
SCG1_MOUSE	SCG1_HUMAN	6.705967162	6.63797068	0.067996482	6.682502003	6.599388642	0.083113361	0.314999242	64	0.503998788
SYN2_MOUSE	SYN2_HUMAN	6.705840426	6.639728108	0.066112319	6.665451998	6.642267998	0.023184001	0.314999242	64	0.503998788
VAMP1_MOUSE	VAMP1_HUMAN	6.692577739	6.639282788	0.053294951	6.705079258	6.599280779	0.105798479	0.314999242	64	0.503998788
5HT2A_MOUSE	5HT2A_HUMAN	6.568370956	6.637064171	-0.068693214	6.553040736	6.593200261	-0.040159525	0.352681374	37	0.54027785
5HT2C_MOUSE	5HT2C_HUMAN	6.779965882	6.617881626	0.162084255	6.704476272	6.662549592	0.04192668	0.352681374	63	0.54027785
SCG3_MOUSE	SCG3_HUMAN	6.700351365	6.634419247	0.065932119	6.723612853	6.630402298	0.093210555	0.393048128	62	0.577540107
SYN1_MOUSE	SYN1_HUMAN	6.683183539	6.637549289	0.04563425	6.703830712	6.661134601	0.042696111	0.393048128	62	0.577540107
CASP1_MOUSE	CASP1_HUMAN	6.483759288	6.609108795	-0.125349507	6.572146563	6.757452755	-0.185306192	0.435872177	39	0.581162903
HOME3_MOUSE	HOME3_HUMAN	6.664728478	6.630986874	0.033741604	6.688520434	6.63604446	0.052475974	0.435872177	61	0.581162903
5HT2B_MOUSE	5HT2B_HUMAN	6.635941749	6.556360094	0.079581655	6.57471335	6.342064077	0.232649273	0.435872177	61	0.581162903
SYT2_MOUSE	SYT2_HUMAN	6.698685156	6.625545694	0.073139462	6.822004847	6.602845919	0.219158927	0.435872177	61	0.581162903
VAMP2_MOUSE	VAMP2_HUMAN	6.662564243	6.631208205	0.031356038	6.664132641	6.593737899	0.070394742	0.435872177	61	0.581162903
BDNF_MOUSE	BDNF_HUMAN	6.558737095	6.634581408	-0.075844313	6.557692466	6.602752264	-0.045059798	0.481250947	40	0.597414969
7B2_MOUSE	7B2_HUMAN	6.630363025	6.634047965	-0.00368494	6.672758316	6.61735007	0.055408246	0.481250947	60	0.597414969
SNP25_MOUSE	SNP25_HUMAN	6.613590235	6.635603458	-0.022013223	6.62043475	6.659422705	-0.038987955	0.481250947	40	0.597414969
GSK3B_MOUSE	GSK3B_HUMAN	6.697491003	6.639190727	0.058300276	6.692481094	6.629793727	0.062687367	0.481250947	60	0.597414969
MK01_MOUSE	MK01_HUMAN	6.6692705	6.642169654	0.027100846	6.679417123	6.636983695	0.042433428	0.578741692	58	0.683104948
NCAM1_MOUSE	NCAM1_HUMAN	6.662446019	6.635884377	0.026561642	6.677624391	6.568919936	0.108704456	0.578741692	58	0.683104948
NLGN3_MOUSE	NLGN3_HUMAN	6.670278702	6.635645096	0.034633607	6.658317361	6.650424676	0.007892685	0.578741692	58	0.683104948
NRX1A_MOUSE	NRX1A_HUMAN	6.590964391	6.631546138	-0.040581748	6.611393301	6.589629057	0.021764245	0.630528914	43	0.732227126
CY561_MOUSE	CY561_HUMAN	6.665459627	6.620104128	0.045355499	6.645972114	6.589652884	0.05631923	0.739364351	55	0.831784895
5HT1A_MOUSE	5HT1A_HUMAN	6.668250635	6.633286522	0.034964113	6.596754911	6.573318384	0.023436527	0.739364351	55	0.831784895
VMAT2_MOUSE	VMAT2_HUMAN	6.571977891	6.556217299	0.015760592	6.542722603	6.433296494	0.109426109	0.795936262	54	0.868294104
SYT1_MOUSE	SYT1_HUMAN	6.656498354	6.639875546	0.016622808	6.699260028	6.64481846	0.054441568	0.795936262	54	0.868294104
5HT6R_MOUSE	5HT6R_HUMAN	6.664842593	6.630664569	0.034178025	6.690838605	6.662842176	0.02799643	0.853428305	53	0.90362997
MK03_MOUSE	MK03_HUMAN	6.671984278	6.631011221	0.040973057	6.663354028	6.679610402	-0.016256375	0.853428305	53	0.90362997
GFAP_MOUSE	GFAP_HUMAN	6.393549024	6.473122954	-0.07957393	6.32286105	6.420975369	-0.098114319	0.911797181	48	0.924639395
HOME1_MOUSE	HOME1_HUMAN	6.619211897	6.636900437	-0.01768854	6.643369931	6.600821984	0.042547947	0.911797181	52	0.924639395
GRIA1_MOUSE	GRIA1_HUMAN	6.604367029	6.626923435	-0.022556406	6.627876307	6.600821961	0.027054345	0.911797181	48	0.924639395
S10AA_MOUSE	S10AA_HUMAN	6.465513913	6.565669457	-0.100155544	6.481464735	6.432970307	0.048494428	0.97051246	49	0.97051246

Table S1. Differential expression analysis of mouse hippocampal qPCR data

Rat protein ID	Human protein ID	case mean expression	control mean expression	Mean log2 fold change	case median expression	control median expression	Median log2 fold change	Wilcoxon p-value	Wilcoxon test statistic	BH-corrected p-value
GRIK4_RAT	GRIK4_HUMAN	7.2471367	6.640307232	0.606829468	7.224257567	6.605678763	0.618578804	0.00952381	24	0.126984127
GRIK5_RAT	GRIK5_HUMAN	7.100129149	6.640725777	0.459403372	7.024202566	6.652483644	0.371718922	0.00952381	24	0.126984127
HOME3_RAT	HOME3_HUMAN	7.566331855	6.637354689	0.928977166	7.559522025	6.596872784	0.962649241	0.00952381	24	0.126984127
GRM1_RAT	GRM1_HUMAN	7.735462254	6.6158921	1.119570154	7.715855873	6.707540763	1.00831511	0.00952381	24	0.126984127
GRM5_RAT	GRM5_HUMAN	7.136055576	6.63350289	0.502552686	7.095618305	6.624651556	0.470966748	0.00952381	24	0.126984127
GRM7_RAT	GRM7_HUMAN	7.090789849	6.642439402	0.448350447	7.075325054	6.643538005	0.431787049	0.00952381	24	0.126984127
5HT1B_RAT	5HT1B_HUMAN	7.175815301	6.58814649	0.587668811	7.147621876	6.668175869	0.479446007	0.017316017	28	0.138528139
CCG2_RAT	CCG2_HUMAN	6.896296745	6.639965381	0.256331365	6.897963003	6.58044702	0.317515984	0.017316017	28	0.138528139
CCG6_RAT	CCG6_HUMAN	6.927932641	6.628243256	0.299689386	6.918579392	6.645298164	0.273281228	0.017316017	28	0.138528139
CCG8_RAT	CCG8_HUMAN	6.927932641	6.628243256	0.299689386	6.918579392	6.645298164	0.273281228	0.017316017	28	0.138528139
KPCA_RAT	KPCA_HUMAN	7.106959146	6.607977629	0.498981516	7.14856131	6.635173947	0.513387363	0.03030303	27	0.181405896
NEB2_RAT	NEB2_HUMAN	7.408614172	6.602267928	0.806346244	7.591957233	6.619119511	0.972837721	0.03030303	27	0.181405896
MTOR_RAT	MTOR_HUMAN	7.028970713	6.607391888	0.421578824	6.996614715	6.765534746	0.231079968	0.031746032	23	0.181405896
SYN3_RAT	SYN3_HUMAN	7.156725269	6.611101377	0.545623892	7.17292749	6.545350645	0.627576845	0.031746032	23	0.181405896
KCC2B_RAT	KCC2B_HUMAN	6.957242576	6.625413942	0.331828634	7.007228211	6.76420813	0.243020081	0.051948052	26	0.244461421
GSK3B_RAT	GSK3B_HUMAN	7.322474947	6.436075126	0.886399821	7.160097064	6.646738698	0.513358366	0.051948052	26	0.244461421
SYPH_RAT	SYPH_HUMAN	7.15368146	6.567482934	0.586198526	7.214794907	6.622051819	0.592743087	0.051948052	26	0.244461421
EAA3_RAT	EAA3_HUMAN	6.929478719	6.613526171	0.315952548	6.966939362	6.568336182	0.39860318	0.082251082	25	0.365560366
BDNF_RAT	BDNF_HUMAN	6.25757159	6.62336324	-0.365791651	6.193771743	6.532940288	-0.339168545	0.093692619	4.5	0.380952381
NTRK2_RAT	NTRK2_HUMAN	7.334768585	6.508699157	0.826069428	7.317412614	6.501439145	0.815973469	0.095238095	21	0.380952381
GRM4_RAT	GRM4_HUMAN	6.944009832	6.619744129	0.324265703	7.014019499	6.652486495	0.361533004	0.177489177	23	0.567965368
GRM8_RAT	GRM8_HUMAN	6.950485396	6.611460306	0.339025091	6.986409141	6.5360529	0.45035624	0.177489177	23	0.567965368
NMDE3_RAT	NMDE3_HUMAN	6.945075563	6.569236536	0.375839027	6.828239247	6.454504938	0.373734309	0.177489177	23	0.567965368
PICK1_RAT	PICK1_HUMAN	7.064895515	6.611988895	0.452906621	7.168450605	6.5360529	0.632397705	0.177489177	23	0.567965368
SHAN1_RAT	SHAN1_HUMAN	7.054756728	6.617880107	0.436876621	7.206284923	6.653919873	0.55236505	0.177489177	23	0.567965368
EF2K_RAT	EF2K_HUMAN	6.901550402	6.483007798	0.418542604	6.944765291	6.197708158	0.747057133	0.246753247	22	0.616883117
EF2_RAT	EF2_HUMAN	7.009599964	6.503516775	0.506083189	6.999508485	6.704595348	0.294913137	0.246753247	22	0.616883117
GRM2_RAT	GRM2_HUMAN	6.779772513	6.556227877	0.223544635	6.854845636	6.373300197	0.481545439	0.246753247	22	0.616883117
NMDE2_RAT	NMDE2_HUMAN	6.886113176	6.606437645	0.279675531	7.08480829	6.668175869	0.416632421	0.246753247	22	0.616883117
NMDE4_RAT	NMDE4_HUMAN	6.94407749	6.566701484	0.377376006	6.906098683	6.578938713	0.327159969	0.246753247	22	0.616883117
DLG4_RAT	DLG4_HUMAN	7.024914079	6.518313237	0.506600841	7.121015029	6.87282876	0.248186269	0.246753247	22	0.616883117
SYN1_RAT	SYN1_HUMAN	6.832207926	6.64047475	0.191733176	6.914886139	6.633721813	0.281164326	0.246753247	22	0.616883117
CCG3_RAT	CCG3_HUMAN	7.280232457	6.622266656	0.657965801	7.24254955	6.709046867	0.533502682	0.257142857	18	0.623376623
AKT1_RAT	AKT1_HUMAN	6.862359689	6.619486749	0.242872941	6.910587031	6.456149035	0.454437997	0.329004329	21	0.634920635
AKT2_RAT	AKT2_HUMAN	6.823943469	6.62427784	0.199665628	6.831020393	6.729280846	0.101739547	0.329004329	21	0.634920635
CNIH3_RAT	CNIH3_HUMAN	6.901454271	6.552850189	0.348604083	6.780308523	6.633721813	0.14658671	0.329004329	21	0.634920635
EAA2_RAT	EAA2_HUMAN	6.98077694	6.520447932	0.460329008	6.933088398	6.482202926	0.450885472	0.329004329	21	0.634920635
GSK3A_RAT	GSK3A_HUMAN	6.963728096	6.552925539	0.410802557	6.957262175	6.834154981	0.123107193	0.329004329	21	0.634920635
NMDE1_RAT	NMDE1_HUMAN	6.953411631	6.572685879	0.380725752	6.954679361	6.813781191	0.14089817	0.329004329	21	0.634920635
NMD3A_RAT	NMD3A_HUMAN	6.800868293	6.622682629	0.178185664	6.789778183	6.658211483	0.131566701	0.329004329	21	0.634920635
RED1_RAT	RED1_HUMAN	6.808482666	6.572606073	0.235876593	6.843961193	6.357552005	0.486409189	0.428571429	20	0.634920635
KCC2A_RAT	KCC2A_HUMAN	6.897311314	6.547838987	0.349472327	6.980381094	6.627898616	0.352482478	0.428571429	20	0.634920635
MK01_RAT	MK01_HUMAN	6.764224094	6.627828195	0.136395899	6.750196884	6.610286657	0.139910227	0.428571429	20	0.634920635
GRIA3_RAT	GRIA3_HUMAN	7.025228181	6.581224954	0.444003227	7.0645928	6.596935142	0.467657657	0.428571429	20	0.634920635
HOME1_RAT	HOME1_HUMAN	6.886201544	6.609108761	0.277092783	6.853372108	6.861707287	-0.008335178	0.428571429	20	0.634920635
NMDZ1_RAT	NMDZ1_HUMAN	6.763658041	6.548065279	0.215592762	7.190316434	6.790511412	0.399805022	0.428571429	20	0.634920635
SHAN2_RAT	SHAN2_HUMAN	6.752777104	6.631209937	0.121567167	6.893246112	6.586464526	0.306781586	0.428571429	20	0.634920635
SHAN3_RAT	SHAN3_HUMAN	6.882368623	6.584888039	0.297480585	7.050922196	6.58345891	0.467463286	0.428571429	20	0.634920635
SYN2_RAT	SYN2_HUMAN	6.824942271	6.629312062	0.19563021	6.78907429	6.653919873	0.135154417	0.428571429	20	0.634920635
STX1A_RAT	STX1A_HUMAN	6.774909117	6.625470007	0.14943911	6.87171667	6.662490364	0.209226306	0.428571429	20	0.634920635
VAMP2_RAT	VAMP2_HUMAN	6.750612345	6.62897243	0.121639915	6.810884579	6.572889668	0.23799491	0.428571429	20	0.634920635
VEGFA_RAT	VEGFA_HUMAN	6.784759323	6.628067565	0.156691758	6.916207683	6.503030646	0.413177037	0.428571429	20	0.634920635
VGFR1_RAT	VGFR1_HUMAN	6.809724051	6.599829138	0.209894913	6.729280205	6.687900522	0.041379683	0.428571429	20	0.634920635
CCG4_RAT	CCG4_HUMAN	6.805643399	6.585909547	0.219733852	6.780030853	6.525129251	0.254901602	0.428571429	20	0.634920635
KAPCA_RAT	KAPCA_HUMAN	6.673523857	6.642252189	0.031271668	6.805414383	6.651105253	0.15430913	0.476190476	16	0.692640693
MK03_RAT	MK03_HUMAN	6.722618959	6.631303319	0.09131564	6.803387662	6.673839056	0.129548606	0.536796537	19	0.740409016
GRIA4_RAT	GRIA4_HUMAN	6.84888367	6.458101017	0.390782652	6.906891597	6.395748328	0.511143269	0.536796537	19	0.740409016
GRM3_RAT	GRM3_HUMAN	6.696270894	6.61968118	0.076589714	6.836381717	6.787902559	0.048479158	0.536796537	19	0.740409016
5HT2B_RAT	5HT2B_HUMAN	6.678973391	6.571028882	0.10794451	6.577428828	6.574486907	0.002941921	0.555555556	13	0.753295669
AKT3_RAT	AKT3_HUMAN	6.865499817	6.633505551	0.231994267	6.882057403	6.617651119	0.264406284	0.583024912	18	0.777366549
5HT1A_RAT	5HT1A_HUMAN	6.634061555	6.524731251	0.109330304	6.592933273	6.395748328	0.197184944	0.662337662	18	0.779220779
5HT5A_RAT	5HT5A_HUMAN	6.725719381	6.622975485	0.102743896	6.765090373	6.539158811	0.225931562	0.662337662	18	0.779220779
GRIK2_RAT	GRIK2_HUMAN	6.689016471	6.372207978	0.316808493	6.670089178	6.504620392	0.165468786	0.662337662	18	0.779220779
GRIK3_RAT	GRIK3_HUMAN	6.72650271	6.372207978	0.354294732	7.003857051	6.504620392	0.499236658	0.662337662	18	0.779220779
GRIP1_RAT	GRIP1_HUMAN	6.690678039	6.570545066	0.120132973	6.867570674	6.680886921	0.186683753	0.662337662	18	0.779220779
S10AA_RAT	S10AA_HUMAN	6.63815859	6.479128722	0.159029869	6.556854807	6.957682486	-0.400827679	0.662337662	18	0.779220779
SNP25_RAT	SNP25_HUMAN	6.539685894	6.640224322	-0.100538428	6.597179906	6.666756592	-0.069576686	0.662337662	12	0.779220779
VGFR2_RAT	VGFR2_HUMAN	6.470390727	6.592878452	-0.122487726	6.452006805	6.659639187	-0.207632382	0.662337662	12	0.779220779
5HT6R_RAT	5HT6R_HUMAN	6.599848035	6.599588155	0.000259881	6.6043679	6.559185866	0.045182034	0.69047619	15	0.800552105
GRIK1_RAT	GRIK1_HUMAN	6.760289558	6.637041452	0.123248106	6.662631643	6.672436435	-0.009804792	0.761904762	14	0.870748299
5HT3A_RAT	5HT3A_HUMAN	6.63136721	6.479509728	0.151857482	6.58340871	6.592457037	-0.009048327	0.792207792	17	0.88023088
GRM6_RAT	GRM6_HUMAN	6.679669245	6.446970708	0.232698537	6.681108908	6.817623258	-0.13651435	0.792207792	17	0.88023088
EAA1_RAT	EAA1_HUMAN	6.576509961	6.539552228	0.036957732	6.689426483	6.697662633	-0.008236151	0.930735931	16	0.992784993
GRIA1_RAT	GRIA1_HUMAN	6.667170969	6.591099051	0.076071917	6.563781175	6.569855608	-0.006074433	0.930735931	16	0.992784993
HOME2_RAT	HOME2_HUMAN	6.659004751	6.576819001	0.08218575	6.763787373	6.287250643	0.47653673	0.930735931	16	0.992784993
5HT2A_RAT	5HT2A_HUMAN	6.480748835	6.428018849	0.052729986	6.430633616	6.672425342	-0.241791726	1	15	1
CNIH2_RAT	CNIH2_HUMAN	6.599163821	6.546114375	0.053049447	6.434835475	6.540709263	-0.105873788	1	15	1
GRIA2_RAT	GRIA2_HUMAN	6.447446742	6.572116079	-0.124669337	6.415263545	6.38024459	0.035018955	1	15	1
NGF_RAT	NGF_HUMAN	6.568151999	6.591073183	-0.022921184	6.501241389	6.683696454	-0.182455065	1	15	1
KPCD_RAT	KPCD_HUMAN	6.636404471	6.554876749	0.081527722	6.84160259	6.725195817	0.116406773	1	15	1

Table S2. Differential expression analysis of rat frontal cortex qPCR data

Transcription Factors	Signal Transduction/ Growth Factors	Synaptic Plasticity/ Scaffolding	Synaptic Vesicles/ Release Machinery	Receptors/Channels/ Transporters	Other
Creb1	*Bdnf*	*Akap9*	*Chgb*	*Cacng2*	*B2m*
Egr1	*Camk2a*	*Arc*	*Rab3a*	*Cacng3*	*Casp1*
Fmr1	*Gsk3b*	*Dlg1*	*Scg3*	*Cacng4*	*Dnm2*
Fos	*Igf1*	*Dlg4*	*Snap25*	*Cacng8*	*Epn1*
Jun	*Jak2*	*Homer1*	*Syn1*	*Esr1*	*Epn2*
Mecp2	*Mapk1*	*Ncam1*	*Syp*	*Esr2*	*Epn3*
Nfkb1	*Mapk3*	*Nlgn1*	*Vamp1*	*Gabra1*	*Gad1*
Rela	*Mapk9*	*Nlgn2*		*Gabra2*	*Gad2*
	Mtor	*Nlgn3*		*Gabra4*	*Gapdh*
	Ngf	*Nrxn1*		*Gabra5*	*Gfap*
	Ntf3	*S100a10 (P11)*		*Gabrb2*	*Map2*
	Ntrk2	*Shank1*		*Gabrd*	*Park2*
	Pik3r1			*Gabrg2*	*Pgr*
	Ppp1r9b			*Gria1*	*Ppia*
	Prkaca			*Gria3*	*Psen1*
	Prkca			*Gria4*	*Rpl13a*
				Grik1	*Rp1p0*
				Grik2	*S100b*
				Grik4	*Sema4c*
				Grik5	*Sema4f*
				Grin1	*Sema4g*
				Grin2a	*Sh3gl1*
				Grin2b	*Sh3gl2*
				Grm1	*Sh3gl3*
				Htr1a	*Tbp*
				Htr1b	*Ubc*
				Htr1d	
				Htr2a	
				Htr2b	
				Htr2c	
				Htr3a	
				Htr4	
				Htr5a	
				Htr6	
				Htr7	
				Slc1a1	
				Slc1a2	
				Slc1a3	
				Slc1a6	
				Slc6a2	
				Slc6a3	
				Slc6a4	

Table S3. List of targets examined in OpenArray study
