# Supplementary material for: Neuroplasticity pathways and protein-interaction networks are modulated by vortioxetine in rodents
Source: BMC Neurosci. 2017 Aug 4;18:56. doi: 10.1186/s12868-017-0376-x (PMC5543755; doi:10.1186/s12868-017-0376-x)
Supplement: Supplementary file 2 — Additional file 2: Figure S1. Merged mouse and rat network (mapped to human proteins) and summary of biological functions of each sub-network. Biological functions were manually extracted from the Function and Gene Ontology fields of the UniProt protein entries. The genes with dark, bold borders were used to build the network of protein–protein interaction partners. Squares with bold borders represent upregulated targets from the rat network, and circles with bold borders indicate differentially-regulated targets from the mouse network. The arrowheads indicate the common targets found in mouse and rat networks. This network of physically-interacting proteins containing clusters related to synaptic plasticity, synaptic transmission, neurodevelopment, cell growth, metabolism, and apoptosis, was significantly modulated in both mouse and rat. [file 12868_2017_376_MOESM2_ESM.pdf]

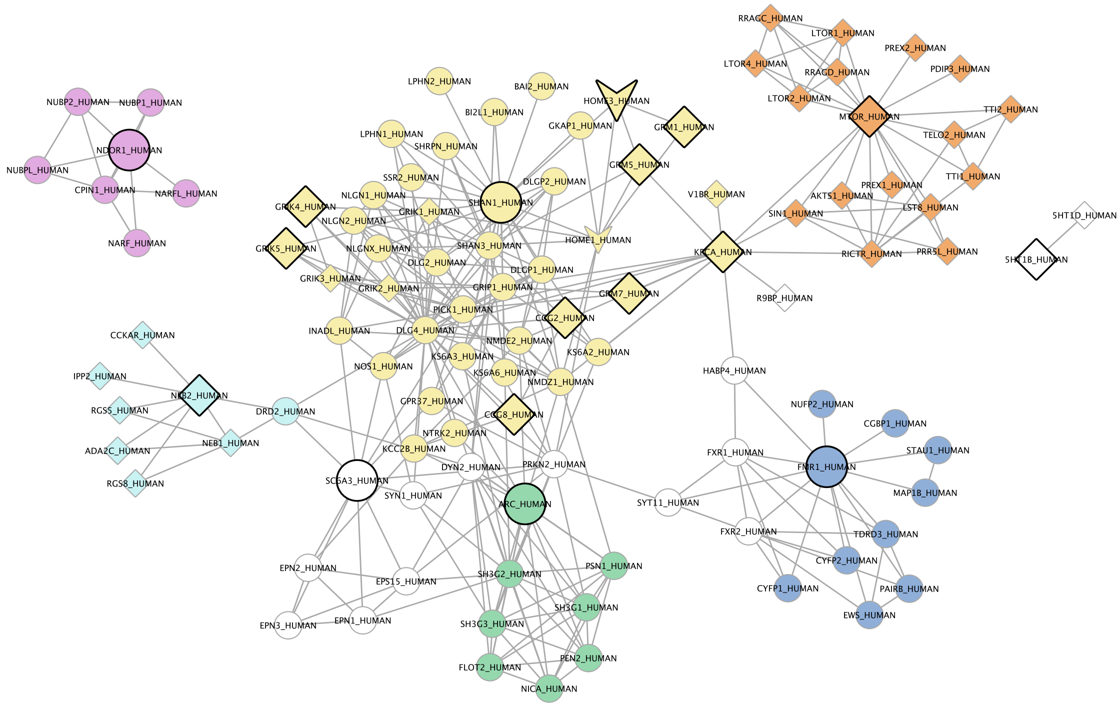

Activation of TOR and mTORC1 signaling cascade, regulation of cell growth and survival

Axon growth, dendrite development, central nervous system development

Notch signaling, central nervous development, pre/postsynaptic endosome recycling

Neurite formation and synaptic transmission through G protein-coupled receptor signaling

Iron-sulfur cluster assembly, small molecule metabolic processes, cell death/apoptosis, cell growth

Synaptic transmission and plasticity, learning and memory, G protein-coupled receptor signaling, neuronal cell-cell interactions, neurodevelopment, migration, neuropeptide signaling pathways, brain specific angiogenesis inhibition, dopamine receptor signaling

Figure S1. Merged mouse and rat network (mapped to human proteins) and summary of biological functions of each sub-network
